# Ambivalence model of suicidality [ABS-model]: an orientation model for the treatment of suicidal individuals

**DOI:** 10.3389/fpsyt.2024.1449565

**Published:** 2024-11-29

**Authors:** Tobias Teismann, Peter C. Britton, Thomas Forkmann

**Affiliations:** ^1^ Mental Health Research and Treatment Center, Ruhr-University Bochum, Bochum, Germany; ^2^ Center of Excellence for Suicide Prevention, VA Finger Lakes Health Care System, Canandaigua, NY, United States; ^3^ Department of Psychiatry, University of Rochester Medical Center, Charleston, SC, United States; ^4^ Department for Clinical Psychology and Psychotherapy, University of Duisburg-Essen, Essen, Germany

**Keywords:** suicidality, ambivalence, risk assessment, case conceptualization, psychotherapy

## Abstract

The existing models for understanding suicidal ideation and behavior do not provide satisfactory orientation for clinical-therapeutic work with suicidal clients. Based on the observation that ambivalence accompanies the entire suicidal process and building on the empirical knowledge about suicidal ambivalence, this article presents the ambivalence model of suicidality (ABS model), a new clinical working model that aims to provide a framework for risk assessment, case conceptualization and treatment planning in the treatment of suicidal individuals. The model divides the suicidal process into three phases (uncertainty phase, transition phase and action phase), describes the psychological state within the different phases, and identifies phase-specific therapeutic interventions. The ABS model is a descriptive model that can be used to structure and organize crisis intervention and psychotherapy with suicidal patients.

## 1 Introduction

Suicidal ideation and behaviour are very common in clinical populations (1). The intensity of suicidal ideation is subject to considerable fluctuations (2). While suicidal ideation does not lead to suicidal behaviour in the vast majority of those affected (3), people who have attempted suicide generally report very rapid transitions from suicidal ideation to suicidal behaviour (4, 5). The considerable dynamics of suicidal ideation and behaviour - in addition to other factors (6) - make it incredibly difficult to reliably assess a person’s suicide risk (7, 8). In addition, a wide variety of constellations of stress factors can lead to suicidal behaviour (9) and suicidal experience and behaviour is therefore motivated very differently in individual cases (10). Treatment approaches for suicidal patients must do justice to this ‘individuality’ of suicidal ideation and behaviour (11).

In this sense, a model of suicidal ideation and behaviour is required, which on the one hand provides clinicians with a framework of understanding and orientation for the exploration and treatment of suicidal patients in clinical practice and on the other hand can be used as an explanatory model for patients in the context of psychoeducation (12). Therapy-related elaborations of common theoretical models of suicidal ideation and behaviour - such as the Interpersonal Theory of Suicidal Behaviour (ITS; 13), the Integrative Motivational-Volitional Model of Suicidal Behaviour (IMV model; 14), or the 3-Steps-Theory (3-ST; 15) - have not yet been developed. All three models can be credited with inspiring research activity and significantly expanding our knowledge of suicidal ideation and behavior. However, it can be questioned whether these models can provide a sufficient framework for individualised clinical-therapeutic work with suicidal individuals due to their focus on individual, very specific factors, that may not resonate with every suicidal person. Another concern is that these models are primarily unidirectional, representing the factors that propel an individual towards suicidal behavior, with minimal consideration of the countervailing factors that protect against it.

Against this background, this article presents a clinical working model that aims to provide a framework for risk assessment, case conceptualization and treatment planning in the treatment of suicidal individuals. At the core of the model stands the observation that ambivalence towards suicide/death accompanies the entire suicidal process of many affected individuals (16): Suicidal individuals report an internal struggle between a wish to die and a wish to live before, during and after suicidal behaviour (e.g., 17–19). Suicide ambivalence offers a therapeutic starting point in different phases of the suicidal process (20, 21) and by exploring the “internal suicide debate” (22), – with intent to “resolve” it towards living (23) –, both individually significant risk and protective factors come into view. In this sense, a focus on suicide ambivalence not only serves to motivate those affected to take a first step back from acting on a suicide wish (20, 23–26), but also informs case conceptualization and treatment planning (cf. 11).

**Figure 1:**
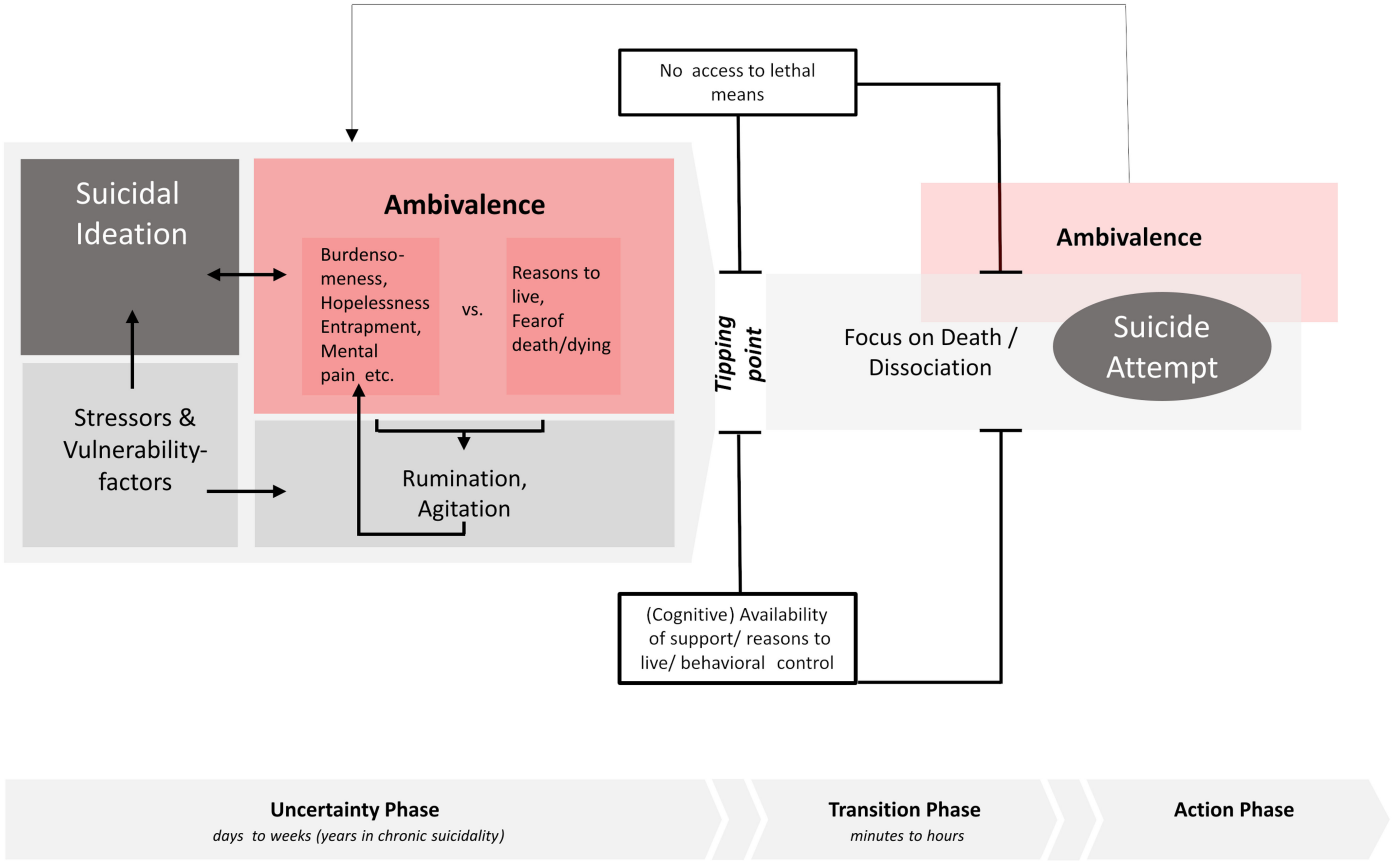
Ambivalence model of suicidality [ABS model].

The ambivalence model of suicidality (ABS model) divides the suicidal process into three phases: the uncertainty phase, the transition phase and the action phase (see **Figure 1**).

The model focuses on the process leading up to a suicide attempt (and not a suicide). This focus is due to the fact that most of our empirical knowledge about suicidal acts comes from the study of individuals who have survived a suicide attempt and are thus able to participate in research and provide insight into the suicidal process. It is unclear whether these findings can be equally applied to individuals who have died by suicide. In the following, the suicidal process is described in relation to the three phases and phase-specific information on (therapeutic) interventions are provided.

## 2 Uncertainty phase

The possibility of suicide usually comes into focus when individuals are exposed to significant and/or multiple stressors (separation, death, unemployment, financial loss, physical illness, trauma (27);) and/or suffer from a mental disorder (28); in the case of prolonged stress and suicidal thoughts, even minor mood changes may be sufficient to activate a more intensive preoccupation with suicidal desires (12, 29, 30). Given the significance of a suicide decision and the difficulty of enacting suicidal behavior, it is to be expected that those affected will enter a phase of ambivalence. Indeed, 94% of suicidal individuals affirmed to have ever had an internal debate about whether to live or die in a survey study (22). Accordingly, Shneidman (31) refers to ambivalence as a cognitive state “that occurs in almost every case of suicide” (p. 129), and Evans and Farberow (32) emphasize ambivalence as “perhaps the most important psychological concept in our understanding of suicide” (p. 12). Ambivalence refers to the simultaneous existence of mutually exclusive attitudes and action tendencies. The term suicidal ambivalence refers to the fact that reasons/wishes to die (e.g., “I can’t bear the pain any longer”) are experienced simultaneously with reasons/wishes to live (e.g., “I want to be there for my daughter”) or to not die (e.g., “I’m afraid of the pain involved in killing oneself”). Of course, ambivalence can vary greatly in intensity and some individuals may not explicitly express ambivalence, but ambivalence rather reveals itself through the usage of words such as “maybe”, “not now”, and “possibly” when talking about suicidal intentions (33).

Reasons for dying are heterogenous and might be associated with physical health issues, relationship problems, the desire to escape, loneliness, a negative self-perception, hopelessness and financial difficulties (34). Moreover, reasons for dying cover all the perceptions that have been identified as central risk factors in the different suicide theories (cf. 10): *I am a burden and others would be better off if I were dead* (perceived burdensomeness; 13). *I don’t really belong anywhere/I am not important to anyone* (thwarted belongingness; 13). *My situation will never change, it is hopeless* (hopelessness; 35). *I am trapped in a hopeless situation* (entrapment; 14, 36). *I just can’t take the thoughts/memories anymore;* (unbearability; 35). *The feelings are so bad; it’s like physical pain, I can’t stand them any more* (mental pain/psychache; 15, 31). Within the ABS model, no weighting of the different perceptions is specified (cf. 12), allowing for suicidal individuals to share their understanding of which factors (“reasons”) they experience as particularly stressful/important and in this sense are to be understood as personally relevant “suicide drivers” (11).

Reasons to die are countered by reasons to live and/or reasons not to die. Reasons to live include themes such as family/friends (“*I don’t want my husband to think I didn’t love him*”), future plans (“*There are still things on my bucket list*”), enjoyable things (“*I could never go swimming again if I were dead*”), self-image (“*I don’t want anyone to think I was a coward*”) and religion (“*My religion forbids suicide*”; 37). In addition, aspects such as fear of the pain involved in dying, fear of death (13, 38), and/or fear of emerging disabled from a suicide attempt, that is reasons against dying (“*I would so like to be dead, but I don’t dare*”), might also have an equivalent effect on suicidal ambivalence.

The strength of the two sides of suicidal ambivalence can vary to a great deal (39). Furthermore, studies showing considerable fluctuations in suicidal thoughts throughout the day (2, 40–42) suggest that the relative importance of reasons to die and reasons to live might also be subject to rapid changes. Finally, it has to be highlighted that even though it is a good thing, if suicidal individuals are (still) ambivalent rather than leaning towards suicide, the experience of ambivalence can be an exhausting and stressful state (43) and ambivalence in itself can therefore represent internal dissonance that can be an additional stressor (“*I just can’t stand this back and forth in my head anymore*”). There is furthermore a risk that persistent ambivalence contributes to persistent rumination and in consequence, to symptoms of overarousal, sleep problems or agitation that may in turn be associated with increased suicidal thoughts or behavior (44, 45). In addition, persistent ambivalence might also prevent individuals from engaging with life and its challenges, resulting in long-lasting and easily activated (chronic) suicidal ideation. It is therefore important to support suicidal individuals in resolving suicidal ambivalence and/or to step out of a constant engagement with suicidal ambivalence.

The uncertainty phase can last minutes, days, weeks or – in the case of chronic suicidal ideation – even years. However, it seems that there are also people who never enter this phase, but immediately transition to the action phase: As such, Bryan, Allen (46) found that 17% of individuals who attempted suicide did so without ever having suffered from suicidal ideation or having made a suicide plan beforehand (cf. 47). Such cases cannot be understood in the sense of the uncertainty phase. Yet, for some proportion of this group, this may not matter from a therapeutic perspective, as practitioners only get to know these patients after a suicide attempt (if at all).

### 2.1 Therapeutic implications

**Figure 2:**
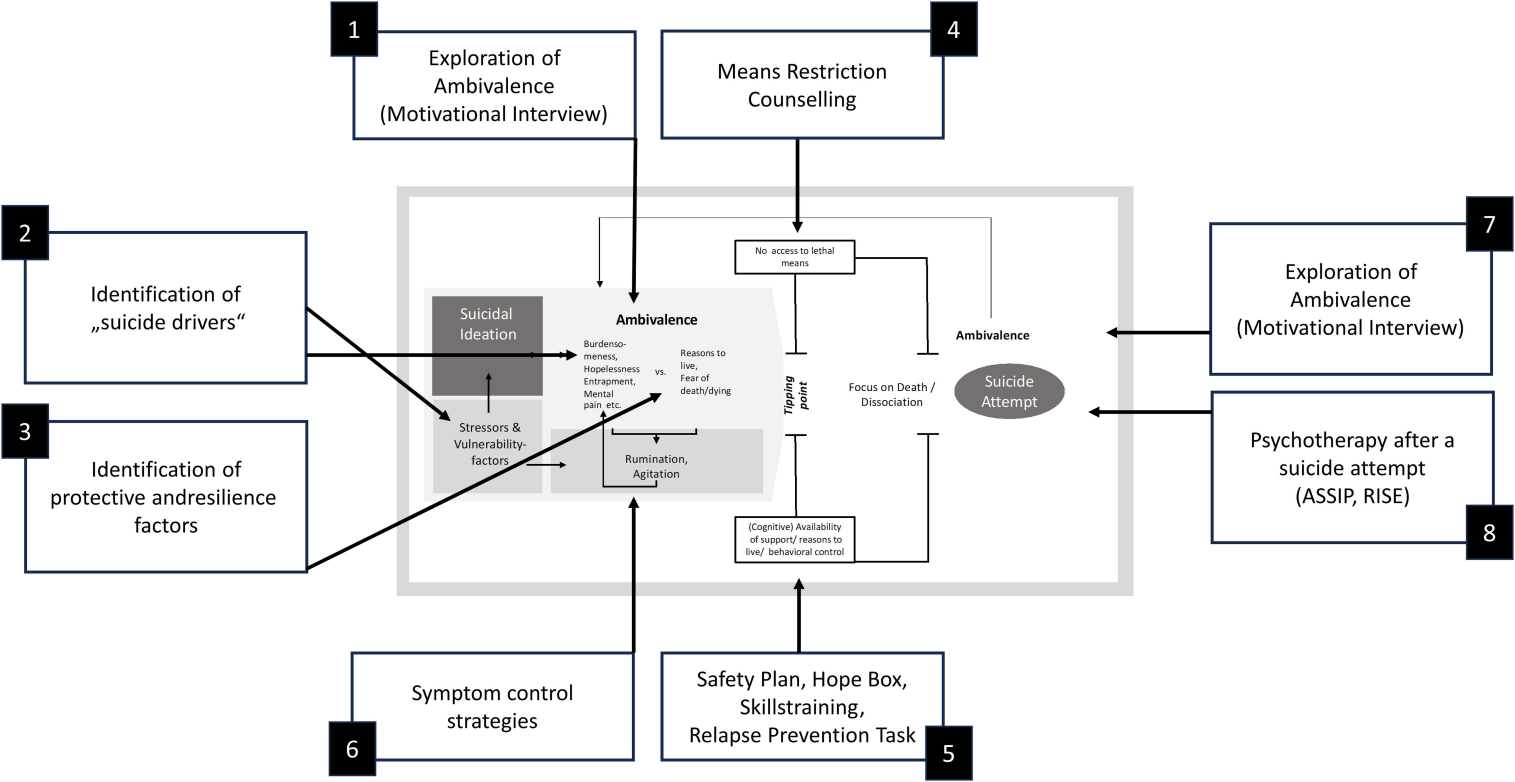
Therapeutic interventions. ASSIP, Attempted Suicide Short Intervention Program (Gysin-Maillart, 2021) (76); RISE, Relapse Intervention after Suicidal Event (Bahlmann et al., 2022) (75).

Therapeutically, it follows from the understanding of the uncertainty phase that both sides of the suicidal ambivalence have to be explored in detail (see **Figure 2**). This pursues two goals: On the one hand, the aim is to get the person into contact with their ambivalence, to empathically validate reasons for dying and to make reasons for living emotionally salient (48). In this way, those affected should be motivated to stay alive (for the time being) and to accept therapeutic support. The literature on Motivational Interviewing with suicidal individuals, for example, gives detailed instructions on how this can be done (20, 23–26, see also 11). On the other hand, the precise exploration of the reasons for/against dying/living serves to come up with a case conceptualization and an individualized treatment plan that targets both the individuals’ personal reasons for dying and reasons for living. A person whose suicidal ideation is driven by perceptions of perceived burdensomeness, for example, possibly needs different therapeutic interventions than a person whose suicidal ideation is driven by perceptions of thwarted belongingness or entrapment (49). In conclusion, the reasons for dying specify what further treatment must focus on. Finally, significant reasons for living define the resources that can be drawn on in the context of crisis management (50).

Ambivalence-focused interventions also aim to contribute to a reduction in (suicide-specific) rumination and, as a result, a reduction in symptoms of overarousal. However, further interventions for dealing with symptoms of rumination/dissociation/insomnia/panic may have to be integrated into a comprehensive treatment plan (12, 49, 51, 52). Finally, it should be noted that stressors and vulnerability factors underlying the individual reasons for dying may also require specific therapeutic attention. In the case of traumatization, trauma therapy may be required to support amelioration of suicidal ideation (53, 54), perfectionism may need to be addressed (55), or individuals have to be supported in making contact with specialized counselling agencies (for example regarding debt, abortion or refugee aid).

## 3 Transition and action phase

With the decision to die by suicide, those affected leave the uncertainty phase and enter the transition phase. The transition phase, therefore, describes suicidal individuals in a state of imminent suicide risk. Whether and when suicidal individuals enter the transition phase cannot be predicted; some authors speak of a “mental accident” in which behavioral control over suicidal impulses (suddenly) fails (56), others speak of coincidence (57) and within dynamic systems approaches (29) the term tipping points is used to characterize the sudden shift into a state of acute suicidality. From qualitative studies, it is known that ambivalence does not have to be resolved in order to enter the transition phase; rather, for some individuals, ambivalence seems to be pushed aside at these moments (58).

Access to lethal means is arguably the single most important factor within the transition to suicidal behavior: Knowledge and availability of lethal means appear to be relevant risk factors for suicidal behavior (59, 60) and restricting access to lethal means is considered one of the most effective methods of suicide prevention (61). A lack of (immediate) availability of lethal means may therefore, on the one hand, protect against entering the transition phase in the first place and, on the other hand, help to prevent a transition to the action phase, i.e., the enactment of suicidal behavior. A second factor that potentially determines whether people enter the transition phase or leave the transition phase unscathed is the (cognitive) availability of (professional/private) supporters, reasons to live and skills in dealing with emotional turmoil (62). Interventions like safety plans (63), crisis response plans (12), hope boxes (64) and skills training (51), all focus on strengthening self-management when dealing with suicidal urges.^1^

Michel (65) highlights the importance of a dissociative state of mind in the immediate run-up to a suicidal act (cf. 66). Suicidal individuals in the transition phase describe an all-embracing “focus on death” (31, 35, 67); to outsiders, the suicidal person may appear “zombie-like” rather than emotionally agitated (68), a “thousand yards stare” (69) seems to characterize some. In general, the transition phase seems to be rather brief: When asked about the time that passed between the decision to die by suicide and the suicidal act, up to 48% of suicide attempters reported a period of less than 10 minutes (5, 70, see also 71). This points to the considerable dynamic of a suicidal crisis.

A peaceful exit from the transition phase is nevertheless possible, however, it often depends on coincidence: the phone rings, the cats scratch at the door, one is approached by a by-stander; or, to put it differently: something happens that “bursts the bubble” (68) the suicidal individual is captured in. Unavailability of lethal means or a sudden reminder of important social partners may also help to live through the transition phase: In such cases suicidal ambivalence might kick in again and a suicide attempt is aborted before the act of killing has been initiated or even shortly after the suicidal act has been initiated. In this sense, 50% of suicide attempters reported in one study that they were still ambivalent during the suicide attempt (18); ambivalence thus seems to accompany the whole suicidal process (72). In this regard, it has to be emphasized that ambivalence does not only refer to a simultaneity of conflicting motives, but can also refer to rapid temporal changes in dominant motives (diachronic ambivalence). It should also be emphasized that the ambivalence experienced at this point in the suicidal process may focus on different questions than during the uncertainty phase. For example, for methods that take time such as overdose, cutting, or hanging individuals may ask “Should I call an ambulance?” or “Should I carry on despite the pain?”. However, the underlying ambivalence for and against dying is potentially composed in the same way as the ambivalence experienced during the uncertainty phase.

### 3.1 Therapeutic implications

Therapeutically, it follows from this understanding of the transition and action phases that suicidal individuals have to become prepared for dealing with strong suicidal impulses and urges: Means restriction counselling (25), safety planning (12, 63), creating a hope box (12, 48), skills training (12, 51) are suitable interventions, in that they support suicidal individuals to establish behavioral control in dealing with suicidal impulses and might prevent them from entering the transition and action phases (see **Figure 2**), and step back into the uncertainty phase.

Interventions in dealing with immediate suicidality are not fundamentally different from strategies used in less acute crisis intervention situations. Still, the starting point is naturally different: the person must first step back from the edge of a tower block, put down a gun, untie a rope; that means a cautious approach and an offer of conversation based on understanding and respect is required. As the conversation progresses, a gentle focus on suicidal ambivalences (21, 73) may then become possible. Initiatives such as the British Samaritans’ “Smalltalks save lives” campaign (www.samaritans.org/support-us/campaign/small-talk-saves-lives/) focus on the fact that cognitive constriction/dissociation (“focus on death”) in the transition phase can also be softened by lay people with the help of small measures – speaking up, being present and showing sympathy (68). It should be noted, that in the case of more severe dissociation, a more resolute approach in order to break this dissociation might be indicated – still, however, based on careful listening and understanding of what the individual needs and respect of his or her personhood.

## 4 Post-attempt phase

In the aftermath of a suicide attempt, 36% to 43% of suicide attempters report that they feel ambivalent about having survived, while 35% are glad to have survived and 14% to 22% regret having survived (17, 74); as such some, but not all individuals will re-enter the uncertainty phase after a suicide attempt. Depending on the individual reaction to survival, a different approach is needed to motivate suicide attempters to come to terms with the suicide attempt, with the prospect of continuing to live, and therefore to prevent future suicidal acts. An ambivalence-friendly approach that takes the experience of shame and stigma into account, psychoeducation and brief therapeutic interventions (75, 76) appears to be suitable for this. In this context, it should be noted that quite a few suicide attempters appear to show signs of post-traumatic stress disorder after a suicide attempt (77) – a low demand for therapeutic treatment of a suicide attempt (78) may therefore also be associated with trauma-related avoidance behavior and must be taken into account therapeutically.

## 5 Discussion

Suicidal ambivalence accompanies the entire suicidal process (31) and offers a therapeutic starting point to motivate suicidal individuals to postpone a decision to die by suicide, to engage in treatment, to establish a different way of dealing with suicidal ideation (20, 23–25) and to explore “suicide drivers” (31) as well as resilience factors. By emphasizing the significance of ambivalence, the ABS model presented here attempts to provide a framework for understanding and treating suicidal individuals. The model does not claim to be able to predict who is at particularly high risk of suicide; in this sense, the model is less explicative than descriptive. As mentioned already, the model furthermore does not assume that every person goes through the described phases in the same way. It should also be clear that the core focus of the model, the experience of ambivalence, is not experienced in the same way by every suicidal person throughout the entire process. Ambivalence is a fluid state that is characterized by inter- and intra-individual variability. Research has shown that suicidal ideation and related risks and warning signs show substantial between- and within subject variance (79). For instance, Hallensleben and colleagues (2) could demonstrate that if measured 10 times a day across seven days, 36% of the variance in active suicidal ideation is accounted for by within-person-variability (see also e.g., 41, 42). It can be inferred that the strength of protective factors may also fluctuate over time, which would mean that suicidal ambivalence (the sum of risk and protective factors) also fluctuates over time. We assume that suicidal ambivalence also shows individual trajectories. It is conceivable that these trajectories differ depending on variables such as age, gender, or cultural backgrounds. The extent to which the assumptions of the model presented here are generally valid independently of age, gender, etc. should be investigated in future studies.

It should also be noted that despite the widespread consensus that ambivalence is a core characteristic of suicidal individuals (31, 32), there is comparatively little high-quality empirical research on suicidal ambivalence (16). The same applies to the description of the mental state of suicidal persons in the transition phase (65). In view of this, various model descriptions of the ABS model must be regarded as provisional. Additional research on the topic of suicidal ambivalence appears warranted. Future studies should aim to verify central assumptions of the model, such as the positive loop between stressors and vulnerability factors, suicidal ideation, ambivalence and rumination/agitation. Prospective studies are of particular interest for this purpose. It would also be important to investigate the trajectories of protective factors and suicidal ambivalence over the course of a suicidal crisis. For all these research endeavors, the use of Ecological Momentary Assessments (EMA) seems particularly promising. EMA allows a repeated assessment of participants in their natural environment (80) and permits capturing moment-to-moment variations in psychological and behavioral variables and calculating relations between the constructs within and across sampling moments. This method has gained much interest in suicidology in recent years (81) and might help to gain a greater understanding of the complex and dynamic interrelations between the variables of the model.

Further, most clinical interventions being studied today address ambivalence in some way, but a comprehensive description of similarities and differences across these interventions, as well as potential implication of the promoted strategies is beyond the scope of this particular article. However, none of this changes the fact that the model parameters can already be used as an orientation framework for the treatment of suicidal patients.

## Data Availability

The original contributions presented in the study are included in the article/supplementary material. Further inquiries can be directed to the corresponding author.

## Footnotes

1 Please note that a detailed description of suicide-specific interventions and treatment manuals is beyond the scope of the present article.
